# Effect of an outpatient copayment scheme on health outcomes of hypertensive adults in a community-managed population in Xinjiang, China

**DOI:** 10.1371/journal.pone.0238980

**Published:** 2020-09-11

**Authors:** Hongpo Yin, Xiaochen Ma, Yanli He, Rujiang Liang, Yongxin Wang, Mei Zhang, Lu Mao, Mingxia Jing

**Affiliations:** 1 Department of Public Health, Shihezi University School of Medicine, Shihezi, Xinjiang, China; 2 China Center for Health Development Studies, Peking University, Beijing, China; London School of Economics and Political Science British Library of Political and Economic Science, UNITED KINGDOM

## Abstract

Hypertension remains the leading risk factor for death and disability in China, and the ability of hypertensive patients to pay for outpatient care and medication has become a critical issue. To report the effect of an outpatient copayment scheme on health outcomes of hypertensive adults in a community-managed population in Xinjiang, we compared changes in outcomes between insured and uninsured groups from baseline to the first follow-up appointment in a community-managed hypertensive population and evaluated these changes based on propensity score matching and the difference-in-difference method. A total of 1,095 individuals in a community-managed hypertension population were selected for investigation at baseline, among which 805 (73.5%) had follow-up data and 749 (68.4%) were included in our analysis. After accounting for the self-reported severity of hypertension and individual characteristics, there were statistically significant improvements in drug treatment of hypertension and self-reported health. We also found increases in drug treatment for hypertension between groups, after correcting for confounding variables (Odds Ratio, OR 8.05, 95% Confidence interval, CI, 1.31–49.35), and in self-reported health between groups after correcting confounders (OR 1.96, 95% CI, 1.12 to 3.42). Adjusted estimates (confounding variables) were corrected for age, sex, income, marital status, education level, employment, family size, self-reported severity of hypertension, course of hypertension, and number of medications. As a result, decreased outpatient copayment was associated with an increase in antihypertensive treatment coverage, and an improvement in self-reported health among community-managed hypertensive populations in Xinjiang, China.

## Introduction

Hypertension remains the leading risk factor for death and disability in China [1, 2]. The prevalence of hypertension among the adult population (aged ≥18 years) in China was 19.8% in 2014, higher than in Korea, Singapore, and the United States [3]. As a consequence, the burden of cardiovascular disease (CVD), in particular stroke, continues to rise in China [4–6]. In 2015, disability-adjusted cumulative life expectancy was worse in China than in Korea, Singapore, and the United States [7, 8]. Throughout middle and old age, controlled blood pressure greatly reduces mortality due to CVD and stroke [9, 10]. Hypertension has a high prevalence rate but remains undertreated and uncontrolled in China [11].

In 2014, the average copay of hypertensive patients in China from the outpatient and pharmacy departments was more than 60% [12, 13]. The annual total cost per capita that year for hypertensive patients was 6,271.80 Chinese yuan (¥) equivalent to 944 US dollars($), and the per capita disposable annual income of Chinese urban households was ¥29,381($4423) [12, 14]. As these statistics show, the ability to pay outpatient department and medication costs has become a critical issue in China, as these costs represented a significant portion of average income [15, 16]. Interventions to increase the ability to pay for antihypertensive treatments, such as health insurance programs, provide financial protection and increase the use of outpatient care and medication [17]. Myriad studies have shown that health insurance improves patient financial security [18]. For example, in the Oregon Health Insurance Experiment, individuals selected by lottery experienced major gains in financial security—a large decrease in medical bills and a virtual elimination of catastrophic out-of-pocket expenses—compared with those who were not selected [19].

For Urban Employee Basic Medical Insurance (UEBMI) in China, the outpatient copayment scheme for eligible patients with chronic diseases, such as hypertension, replaced Medical Savings Accounts (MSAs) [20]. MSAs were primarily used to cover outpatient expenses. However, evidence from China, Singapore, and the United States suggests that MSA schemes are generally inefficient and do not provide adequate financial support to patients in outpatient departments [21]. To lighten the load of outpatient expenses for chronic diseases, the government gradually established an outpatient copayment scheme. The main policy objective of the outpatient scheme was to compensate for the heavier burden of chronic patients with outpatient medication costs through deductibles, coinsurance, and payment ceilings [20]. Patients who receive policy support can be reimbursed when they use outpatient services or purchase medications for hypertension. For example, pursuant to the policy in force in 2016, once a patient’s out-of-pocket and drug-paying total has reached ¥100($15), the patient can enjoy a reimbursement rate of 80% up to a total ceiling of ¥3,000($452) [22]. Once a patient exceeds this outpatient co-payment scheme ceiling, they are required to pay 100% (additional details of the outpatient copayment scheme for hypertension are described in S1 File).

Often, health insurance may help hypertensive patients because long-term treatment is unaffordable [17, 23]. There is strong evidence to indicate that having health insurance leads to increased use of prescription medication as well as better medication adherence [18, 24]. Evidence from ten years of the highest quality research has indicated that health insurance has produced significant, multifaceted, and nuanced benefits to health [18]. However, studies evaluating the relationship between health insurance and health status are scarce, and those in existence have demonstrated conflicting results [25]. This is likely due to the fact that most of these studies have been retrospective and used cross-sectional data [26]. At the same time, the population covered by insurance, the measures of health outcomes, and the length of the study period are different. A study that evaluated the effect of a 2008 Medicaid expansion in Oregon on adults with incomes below 100% of the federal poverty level showed that Medicaid coverage generated no significant improvements in measured physical health outcomes in the first 2 years [19, 27]. In rural Nigeria, increased access to and improved quality of health care through a community-based health insurance (CBHI) program was associated with a significant decrease in blood pressure in a hypertensive population over a span of 5 years [28, 29]. However, the increase in blood pressure in both groups observed between midline and end-line indicated that the effect of insurance was limited. It was difficult to maintain long-term adherence to antihypertensive medication and treatment monitoring for patients with varying characteristics, such as in terms of health literacy.

The 2016 outpatient copayment scheme ceiling rose from ¥2,000($301) to ¥3,000($452) in a region of Xinjiang, China, which decreased outpatient copayments [22]. We hypothesized that insured patients would experience favorable trends in health outcomes and health service usage after the 2016 reform implementation. In the present study, we evaluated the effect of this change to the ceiling in the outpatient copayment scheme on the health of a community-managed hypertensive population in Xinjiang, China.

## Methods

### Study design and population

We used a quasi-experimental design to measure the effects of the outpatient copayment scheme for eligible hypertensive patients (“the intervention”) in blood pressure, health-related quality of life (HRQOL), blood pressure control, drug treatment of hypertension, and self-reported health within a community-managed population. We compared outcomes from baseline at six months after the policy change (June—July 2016) with outcomes at 18 months after the policy change (June—July 2017) between insured and uninsured groups. We also considered the difference in changes from baseline to follow-up between the insured and uninsured groups to depict the intervention effect in Ordinary Least Squares regression models and logistics regression models.

The study population was diagnosed with hypertension by clinical doctors. A follow-up survey was conducted by administering an original questionnaire to five communities at the Eighth Division of Xinjiang Production and Construction Corps, China, from June to July 2016 and from July to August 2017. The authors designed the questionnaire specifically for this study. To be included in the study, individuals had to be (1) hypertensive patients who live long-term in selected communities, (2) insured with the UEBMI, (3) managed by a community health institution, (4) insured by an outpatient copayment scheme before 2016 for eligible patients with hypertension in the insured group, and (5) never insured by an outpatient copayment scheme for eligible patients with hypertension in the uninsured group. Exclusion criteria comprised the following: those who (1) had communication difficulties, (2) exhibited severe cognitive impairment or were diagnosed with severe dementia and mental disorder, (3) were too sick to finish the interview, (4) were uncooperative, (5) passed away, and (6) in the uninsured group applied for the outpatient copayment scheme in 2017 successfully. At baseline, we selected 1,095 community-managed patients with hypertension from five communities in the Eighth Division of Xinjiang Production and Construction Corps, China, between June and July 2016.

### Sampling and sample size

Using a multistage sampling method based on socio-economic status, population size, and reimbursement level within the outpatient copayment scheme for eligible patients in different regions of Xinjiang, we selected five communities in one region for the study sample. The level of reimbursement differs across the regions of Xinjiang. Therefore, it was problematic to select multiple regions for evaluation to reflect the effects of policy. Ultimately, we selected the Eighth Division, a region with a high level of economic development and a dense population. These five communities in the Eighth Division also have strong chronic disease management and services, and they meet the sample size requirements. In 2016, 4/5th of the Eight Division had an urban population while 1/5th had a rural population. [30]. At the same time, considering the communities in rural areas, most of the residents lived near the farms and were sparsely distributed. Therefore, three of the five communities were located in urban areas and the other two communities were rural.

We performed sample-size calculations at a conventional power of (1-beta) 0.80 and alpha of 0.05 (two-sided testing), which revealed that a minimum of 150 participants was required to ensure efficacy. Thus, the calculations confirmed that our sample size was large enough for effective data analysis.

### Data collection

Trained interviewers administered an original questionnaire to collect demographic, socio-economic, clinical, and medical information. The questionnaire asked about health outcomes, including blood pressure, health-related quality of life (HRQOL), blood pressure control, drug treatment of hypertension, and self-reported health. The data were collected through 10–15-minute face-to-face interviews. Blood pressure was measured three times on the upper left arm after at least 10 minutes of rest, using a mercury sphygmomanometer administered by a clinician-trained investigator. We assessed HRQOL using the EQ-5D, a generic measure of HRQOL, which can address these health issues [31]. The EQ-5D-3L consists of five dimensions: mobility, self-care, usual activities, pain/discomfort, and anxiety/depression. Each dimension had three levels of response regarding severity (no problems, moderate problems, and extreme problems). The Chinese utility value set was used to obtain utility scores for EQ-5D health states [32]. Additionally, the EQ-5D included the EQ visual analogue scale (EQ-VAS), which records the patient’s self-rated health on a vertical visual analogue scale, the endpoints of which are labelled “Best imaginable health state” and “Worst imaginable health state.” The EQ-5D and EQ-VAS have been validated in previous studies.

### Ethical review

Ethical clearance was obtained from the ethical review committee of Shihezi University. The study was funded and approved by the National Natural Science Foundation of China (Project Identification Code: 71363047). Participants were assured, via a formal written release or an informed consent statement, that their answers in this study would not impact their subsequent medical benefits. Informed consent was obtained from all participants by signature or by fingerprint on the consent form or written release.

### Data analysis

Blood pressure control (“control”) was defined as a measured systolic blood pressure of less than 140 mm Hg and a measured diastolic blood pressure of less than 90 mm Hg. Drug treatment of hypertension (“treatment”) was defined as individual-linked hypertension medication observed in the medicine cabinet, or the self-reported hypertension medication use portion of the study interview questionnaire [28, 29]. The outcome variable of the difference-in-differences model cannot be an ordered multi-categorical variable [33, 34]. Self-reported health was divided into five levels that represented very good, good, ordinary, poor, and very poor health. In our study, self-reported health was converted into a binary outcome variable, and “very good” and “good” were considered as “self-reported health was good.” The differences in changes in blood pressure from baseline to the first follow-up between the insured and uninsured groups were predefined as the outcome to measure the effect of the program on health before the follow-up survey. Those primary outcomes were defined because the blood pressure of hypertensive patients provides an accurate, objective report of disease biomarkers. The main aspects of the reimbursement of the outpatient copayment scheme that were considered included the cost of antihypertensive drugs. Our hypotheses aimed to identify which components of health status and health service usage would be affected by an insurance program within two years. The differences over time in control, treatment, health-related quality of life (HRQOL), and self-reported health between respondents with hypertension in the insured and those in the uninsured groups constituted secondary outcome measures.

The goal of this analysis was to examine whether or not enrollment in the outpatient copayment scheme for eligible patients with hypertension was associated with decreased blood pressure as compared to similar patients that did not participate in the study during the same time period. A difference-in-differences approach was used, which is an econometric method for evaluating changes in outcomes occurring after the implementation of a policy [28, 35]. This approach isolates an improvement in outcomes related to an intervention (e.g., outpatient copayment scheme for eligible patients with hypertension) that exceeds changes over the same time period in a control group that was not exposed to the intervention.

However, insured individuals may not be representative of all individuals with hypertension. The outpatient copayment scheme for chronic diseases has a series of application conditions, one of the most important of which is the severity of the disease. Therefore, we used two separate strategies to adjust for the potential differences between insured and uninsured groups. First, propensity scores matched the insured and uninsured groups on baseline patient demographics, clinical characteristics, and medication. Second, multivariate adjustment accounted for all observable individuals and patient characteristics that were included in the propensity score model. To ensure that individuals in the insured and uninsured groups were on the same trajectory for severity of hypertension at baseline, they were matched for self-reported hypertension severity, course of hypertension, and number of medications with the uninsured individuals. This matching ensured that the influence of confounding variables and the bias introduced by self-selection into (or out of) the program can be reduced in the pre-enrollment period, which is one of conditions of a difference-in-differences methodology [36, 37].

### Statistical analysis

We analyzed the data using commercially available statistical software (Stata, version 14.0). We examined population characteristics of the participants with hypertension in the insured and uninsured groups, comparing the statistics using the following bivariate analyses: the Kruskal-Wallis test for continuous variables and the chi-square test or Fisher’s exact test for categorical variables.

To create propensity scores for observations matching at baseline, we created a logistic regression model with outpatient medical insurance for hypertension participation (versus not participating) as the dependent variable. Independent variables included age, sex, annual household income, marriage, education, employment, family size, self-reported hypertension severity, course of hypertension, and number of medications. For matching, we used caliper matching with one-to-one matches based on the propensity score without replacement [38].

To perform the difference-in-differences analysis, we used regression models to evaluate the relationship between each dependent variable (systolic blood pressure, diastolic blood pressure, EQ-5D index, EQ-VAS, control of blood pressure, treatment of hypertension, and self-reported health) and enrollment in the outpatient copayment scheme [36, 38]. The regression had prerequisites. Simple linear regression was appropriate when the following conditions were satisfied. First, the dependent variable Y had a linear relationship to the independent variable X. To verify this, we ensured that the XY scatterplot was linear and that the residual plot depicted a random pattern. Second, for each value of X, the probability distribution of Y had the same standard deviation σ. When this condition was satisfied, the variability of the residuals was relatively constant across all values of X, which is easily confirmed in a residual plot. Third, for any given value of X, the Y values are independent, as indicated by a random pattern on the residual plot. A histogram or a dot plot can reveal the shape of the distribution. Here, we used logistic regression for the dichotomous outcomes variables and Ordinary Least Square (OLS) for the continuous variables [33]. Two dummy variables were included. One indicated whether the patient had insurance at baseline or the first follow-up, and another indicated survey times. Finally, an interaction term of the insured (versus the uninsured) variable and a yearly time variable (Insured x Time) was added. The coefficient from these interaction terms, the difference-in-differences estimators, can be interpreted as the independent relationship of enrollment in the outpatient copayment scheme and outcomes for patients with hypertension at those time periods [33, 34].

## Results

### Survey response rate and attrition

**Fig 1:**
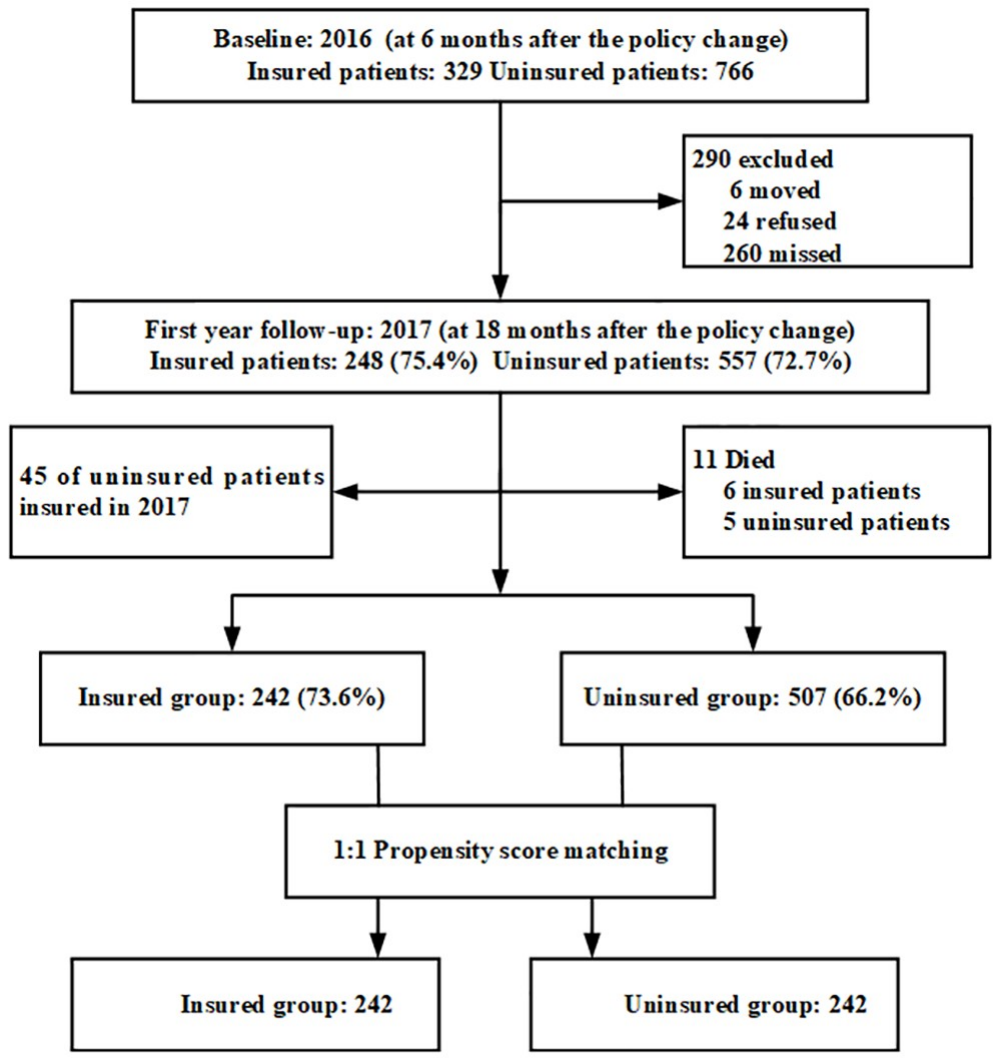
Participation in the 2016 and 2017 surveys and reasons for attrition.

Between July and August of 2016, 1,095 individuals in the community-managed hypertensive population were observed at baseline, including 329 insured and 766 uninsured patients. Between July and August of 2017, 805 (73.5%) individuals were followed up with during the first follow-up survey, including 248 of the insured population and 557 of the uninsured population. A total of 290 individuals were excluded from the first follow-up survey in 2017. Of these, six moved away, 24 refused to cooperate with the survey, and 260 missed their interview appointment. Of the 805 patients who completed follow-ups in 2017, 45 uninsured group members were eligible for insurance, but five patients died, while six members of the insured group died. Longitudinal data were available for 749 individuals (68.4%), of which 242 (73.5%) were in the insured group and 507 (66.2%) were in the uninsured group (Fig 1). Eventually, we obtained matching insured and uninsured groups through the one-to-one propensity score matching method, including 242 from each of the groups (Fig 1).

### Study sample characteristics

Table 1 shows the general characteristics of the insured and uninsured groups before and after propensity score matching at baseline. Insured and uninsured group members were well matched on the variables used in propensity score matching. After matching, self-reported severity of hypertension was 183 (75.62) versus 187 (77.27), p = 0.850, > = 10, of course of hypertension, 175 (72.31) versus 172 (71.07), p = 0.582, > = 3, and of medications, 27 (11.16) versus 19 (7.85), p = 0.462 between the insured and uninsured groups, respectively. While many other characteristics at baseline were similar (age, sex, income, marriage, education, work, family size), even after conducting propensity matching, the uninsured group included slightly more females than the insured group, while the insured group had a slightly higher annual household income than the uninsured group. In order to assess the quality of the matching, we also reported the region of common support, the average treatment effects on the treated (ATT), measures of the balancing of the variables before and after matching, and the density plot of the distribution of the propensity score for treated and untreated before and after matching (further details of the outpatient copayment scheme for hypertension are described in S2 File).

**Table 1:** Characteristics of selected individuals in the insured group compared to the uninsured group before and after propensity score matching at baseline.

Characteristic	Before Propensity Score Match			After Propensity Score Match		
	Uninsured N = 507	Insured N = 242	P-value ^a^	Uninsured N = 242	Insured N = 242	P-value
**Age, median (IQR)**	73.0(68–77)	74(70–77)	0.086	74.5(70–78)	74.0(70–77)	0.569
**Sex, NO, (%)**						
Male	210(41.42)	109(45.04)	0.349	100(41.32)	109(45.04)	0.409
Female	297(58.58)	133(54.96)		142(58.68)	133(54.96)	
**Income, median (IQR)** ^b^	60000(48000–72000)	66000(50400–72000)	0.205	64200(52800–72000)	66000(50400–72000)	0.845
**Marital status, NO, (%)**						
Married	396(78.10)	197(81.40)	0.299	192(79.34)	197(81.40)	0.567
Other	111(21.89)	45(18.60)		50 (20.66)	45(18.60)	
**Education level, NO, (%)** ^c^						
Illiterate	164(32.35)	70(28.93)	0.398	80(34.30)	70(28.93)	0.401
Primary school	168(33.14)	92(38.02)		81(33.47)	92(38.02)	
Junior high school and above	175(34.52)	80(33.06)		78(32.23)	80(33.06)	
**Employed, NO, (%)**						
Retirement	474(93.49)	238(93.35)	0.004	233(96.28)	238(98.35)	0.160
Others	33(6.51)	4(1.65)		9(3.72)	4(1.65)	
**Family members, NO, (%)**						
1	58(11.44)	22(9.09)	0.330	21(8.68)	22(9.09)	0.873
>1	449(88.56)	220(90.91)		221(91.32)	220(90.91)	
**Self-reported disease severity, NO, (%)** ^c^						
Mild	211(41.62)	183(75.62)	0.002	187(77.27)	183(75.62)	0.850
Moderate	294(57.99)	56(23.14)		53(21.90)	56(23.14)	
Severe	2(0.39)	3(1.24)		2(0.83)	3(1.24)	
**Course of hypertension (year), NO, (%)** ^d^						
<5	117(23.08)	15(6.02)		11(4.55)	15(6.02)	
5~10	133(26.23)	52(21.49)	0.000	59(24.38)	52(21.49)	0.582
>=10	257(50.69)	175(72.31)		172(71.07)	175(72.31)	
**Number of medications, NO, (%)**						
0	53(10.45)	8(3.31)	0.000	8(3.31)	8(3.31)	0.462
1~2	434(85.60)	207(85.53)		215(85.54)	207(85.53)	
>=3	20(3.94)	27(11.16)		19(7.85)	27(11.16)	

Abbreviations: IQR, interquartile range.

^a^ indicates differences between the uninsured and insured groups (χ^2^ test or Fisher’s exact test for categorical and Kruskal-Wallis test for continuous variables, *P < 0*.*05*).

^b^ indicates total annual household income.

^c^ indicates self-reported severity of hypertension.

^d^ indicates the length of time that hypertension lasts

### Physiologic measures and health-related quality of life

Table 2 reports the policy effects on blood pressure, the EQ-5D index, and the EQ-VAS between insured and uninsured groups with the difference-in-differences approach. There were no statistically significant decreases in blood pressure in the insured group after the policy changed as compared with the uninsured group: systolic blood pressure (Adjusted Between-Group Difference 0.51, 95% CI, -3.73 to 4.75); diastolic blood pressure (Adjusted Between-Group Difference 0.05, 95% CI, -2.83 to 2.92). There were no statistically significant decreases in HRQOL in the insured group after the policy changed as compared with the uninsured group (EQ-5D index: Adjusted Between-Group Difference 0.02, 95% CI, -0.02 to 0.06; EQ-VAS: Adjusted Between-Group Difference 2.43, 95% CI, -2.11 to 6.98).

**Table 2:** Changes in physiologic measures of health and HRQOL between insured and uninsured groups estimated by difference-in-differences method.

Variable	Uninsured Group (n = 242)		Insured Group (n = 242)		Unadjusted Between-Group Difference		Adjusted Between-Group Difference ^a^	
	6 months after policy change, mean (SD)	18 months after policy change, mean (SD)	6 months after policy change, mean (SD)	18 months after policy change, mean (SD)	Coefficient ^b^ (95% CI),	P-value	Coefficient ^b^ (95% CI)	P-value
**Physiologic measures of health**								
SBP	140.52(16.79)	137.60(18.31)	139.54(16.08)	137.16(16.10)	0.54(-3.71, 4.79)	0.803	0.29(-3.96, 4.54)	0.813
DBP	80.15(13.16)	77.40(10.55)	79.80(11.40)	77.14(10.50)	0.09(-2.80, 2.98)	0.953	-0.06(-2.94, 8.83)	0.975
**Health-related quality of life**								
EQ-5D index	0.89(0.15)	0.88(0.15)	0.843(0.17)	0.84(0.20)	0.01(-0.04, 0.05)	0.781	0.02(-0.02,0.06)	0.306
EQ-VAS	67.43(19.13)	64.25(17.15)	64.198(18.66)	62.51(18.37)	1.49(-3.15, 6.12)	0.530	2.40(-2.15, 8.94)	0.294

Abbreviations: SBP, systolic blood pressure; DBP, diastolic blood pressure SD, Standard Deviation

^a^ Adjusted estimates were corrected for age, sex, income, marital status, education level, employment, family members, self-reported severity of hypertension, course of hypertension, and number of medications.

^b^ The coefficient for the variable reflected the true effect of the intervention.

The R^2^ of model goodness test for adjusted estimates: SBP(0.036), DBP(0.046), EQ-5D index(0.204), EQ-VAS(0.068).

### Health, medications, and self-reported health measures

Table 3 reports the policy effects on controlled hypertension, treatment of hypertension, and self-reported health between insured and uninsured groups from the difference-in-differences approach at the first follow-up. The number of patients with controlled blood pressure increased from 85 (35.12%) to 103 (42.56%) in the insured group. There were no statistically significant decreases in control of blood pressure in the insured group after the policy changed as compared with the uninsured group: control of blood pressure (Adjusted Between-Group Difference OR, 0.84, 95% CI, 0.50 to 1.41). The number of patients with drug treatment for hypertension increased from 236 (97.52%) to 238 (98.34%) in the insured group. But in the uninsured group, it dropped from 235 (97.11%) to 221 (91.32%). Similarly, the number of patients with good self-reported health increased from 70 (28.93%) to 85 (35.12%) in the insured group and decreased from 89 (36.78%) to 74 (30.58%) in the uninsured group. There were statistically significant increases in treatment of hypertension and self-reported health in the insured group at the first follow-up appointment, as opposed to the uninsured group’s treatment of hypertension (Adjusted Between-Group Difference OR 8.05, 95% CI, 1.31 to 49.35) and self-reported health (Adjusted Between-Group Difference OR 1.96, 95% CI, 1.12 to 3.42).

**Table 3:** Changes in health, medications, and self-reported health measures between insured and uninsured groups estimated by the difference-in-differences method.

Variable	Uninsured Group (n = 242)		Insured Group (n = 242)		Unadjusted Between-Group Difference		Adjusted Between-Group Difference ^c^	
	6 months after policy change, N (%)	18 months after policy change, N (%)	6 months after policy change, N (%)	18 months after policy change, N (%)	OR ^d^ (95% CI)	P-value	OR ^d^ (95% CI)	P-value
**Health and medications**								
Control ^a^	94 (38.84%)	131(54.13%)	100(41.32%)	126(52.07%)	0.83(0.50, 1.38)	0.474	0.84(0.50, 1.41)	0.451
Treatment ^b^	235(97.11%)	221(91.32%)	236(97.52%)	238 (98.35%)	4.83(1.03, 22.71)	0.046	7.08(1.17, 42.99)	0.024
**Self-reported measures of health**								
Self-reported health	89 (36.78%)	74 (30.58%)	70(28.93%)	85 (35.12%)	1.76(1.03, 3.01)	0.040	1.97(1.13, 3.44)	0.018

^a^ Control of blood pressure was defined as measured systolic blood pressure of less than 140 mm Hg and measured diastolic blood pressure of less than 90 mm Hg.

^b^ Drug treatment of hypertension was defined as individual-linked hypertension medication observed in the medicine cabinet survey or self-reported.

^c^ Adjusted estimates were corrected for age, sex, income, marital status, education level, employment, family members, self-reported severity of hypertension, course of hypertension, and number of medications.

^d^ The OR for the variable reflected the true effect of the intervention.

The Pseudo R^2^ of model goodness test for adjusted estimates: Control (0.037), Treatment (0.326), Self-reported health (0.044).

## Discussion

Our study showed that the outpatient copayment scheme had no significant effect on systolic blood pressure, diastolic blood pressure, blood pressure control, EQ-5D index, or EQ-VAS, but it increased the coverage of drug treatment for hypertension and led to a substantial improvement in self-reported health. With respect to clinically measured health, this pattern of findings—an improvement in an individual’s recollection of their health status, yet no improvement in their physical health (*See* Table 2)—was mirrored in the self-reported health measures, with improvements concentrated in self-reported health rather than physical health (*See* Table 3). The improvements appeared to be specific to self-reported health measure. In addition, in terms of drug treatment coverage of hypertension and self-reported health, the uninsured group experienced a decline from 2016 to 2017 compared with the insured group. This seemed to indicate that the outpatient copayment scheme had stopped the trend of the insured group’s decline in drug treatment and self-reported health, instead producing a slight increase in these variables. Further, while the outpatient copayment scheme had no significant effect on blood pressure and blood pressure control, the data still depict a clear decline in these variables.

To evaluate the extent to which these improved outcomes were independently associated with enrollment in the outpatient copayment scheme, we matched each insured individual with one control uninsured individual who had similar characteristics and severity of hypertension at baseline. These findings are consistent with previously published results conducted approximately one year after the policy changed. Using the control group of uninsured individuals, the independent association of enrollment in the outpatient copayment scheme with adverse outcomes and the UEBMI payments could be isolated, removing any confounding background trends toward biased outcomes. These findings show that enrollment in the outpatient copayment scheme may improve the self-reported health of hypertensive individuals but may be insufficient to reduce blood pressure in our study population.

Sustained drug treatment for hypertension is vital for prevention of cardiovascular disease (CVD) [39, 40], particularly in China, where the levels of diagnosis, treatment, and control are much lower than in Western populations [3, 11]. It is difficult for patients with hypertension to adhere to antihypertensive drug regimens and uninterrupted quality treatment for longer periods without financial support [41, 42]. For the individuals in the insured group, antihypertensive drugs became more affordable after the ceiling for the outpatient copayment scheme for hypertension rose to ¥3,000($452). This financial support has likely resulted in sustained antihypertensive treatment among insured group members. This sustained drug treatment, accessed through financial support, has likely contributed to the improvement in self-reported health. Based on the data, one can infer that self-reported health may also be affected by individuals’ subjective feelings about the care that they receive. The financial support appears to produce significant benefits to health such as self-reported health. Some benefits may manifest in the psychological well-being born of knowing one can afford care when one gets sick. This modest but cumulative change was called “the heroism of incremental care” [18]. However, with high drug treatment coverage in the insured group, blood pressure and control did not show an effective reduction. This may be due to a drug treatment coverage rate of more than 90% in both groups at baseline. This makes the effect of the drug on blood pressure limited. Therefore, this intervention likely resulted in increasing antihypertensive treatment coverage in the insured group and contributed to the self-reported health improvement observed. These findings suggested that the intervention of the outpatient copayment scheme could help to raise antihypertensive drug treatment coverage.

This study demonstrates that outpatient copayment schemes result in an increase in the use of antihypertensive drugs, indicating better chronic care for patients. We hypothesized that decreased outpatient copayment would result in better quality drugs and greater self-reported health with better chronic care in the insured group compared with the uninsured group. Evidence from ten years of the highest quality research summarized in a review of the literature has indicated that health insurance programs significantly increase family economic security, chronic illness treatment, and access to medications [18]. Those increases appear to have produced significant, multifaceted, and nuanced benefits to health status and mortality [24].

Health insurance programs could significantly increase family economic security, chronic illness treatment, and access to medications [18]. These changes will ultimately help tens of thousands of people live longer lives. However, several studies from other countries have shown that effects on health are insignificant, and the results are conflicting. A study that evaluated the effect of a 2008 Medicaid expansion in Oregon on hypertension, HRQOL, and self-reported health found that insurance led to increased access to antihypertensive treatment and better self-reported health [19]. After an average of 17 months of coverage, there were no statistically significant changes in blood pressure and HRQOL, but those changes would have been considered clinically significant in the Oregon experiment [19]. However, the Oregon study is rarely cited in the evaluation of health insurance programs, and its estimates of the effect of Medicaid coverage on health only apply to able-bodied, uninsured adults with incomes below 100% of the federal poverty level who express interest in insurance. In addition, in the Oregon study, there was likely selection bias among the voluntary group with Medicaid coverage, which limits the value of a comparison between the lottery and control groups [19].

Patients with better health literacy, more health-seeking behavior, and more severe diseases may be more likely to enroll in an insurance program. They may also be more likely to start and adhere to a treatment regimen, independent of their insurance status. For example, in rural Nigeria, the CBHI program was associated with a significant longer-term reduction in blood pressure in a hypertensive population after 5 years of coverage [28]. Although there were significant long-term reductions in blood pressure, an increasing trend in blood pressure in both hypertension respondent groups was observed between midline and end-line compared to lower pressure measurements at baseline. Sustained blood pressure reductions require long-term access and adherence to uninterrupted quality treatment. Even in high-income countries where access to care is guaranteed, it is difficult to motivate patients to adhere to antihypertensive medication and treatment monitoring for a long time [29]. Our own data support this notion because treatment coverage for hypertension in the insured group was lower compared with treatment coverage in the uninsured group at baseline, and patients with more severe hypertension were more likely to apply for the outpatient copayment scheme. The strength of our study results is the elimination of selection bias through the use of propensity score matching and difference-in-differences analyses paired with longitudinal data collection. This analysis compares changes in outcomes over time in insured and uninsured group.

Blood pressure, EQ5D index, VAS, controlled hypertension, treatment of hypertension, and self-reported health constitute a subgroup of the set of health outcomes potentially affected by health insurance [18, 43]. We chose these health outcome variables because they were important to the measurement of health status, they were feasible to measure within the confines of our methods, they were prevalent in the community-managed population isolated for our study, and they were plausibly modifiable by effective treatment in the short term [44]. Nonetheless, our power to detect changes in health was limited by the study design. Indeed, the only health outcome variables in which we detected improvement were in the treatment of hypertension and self-reported health, which were by far the most commonly documented in prior studies [45, 46]. Blood pressure measurements are sensitive: their accuracy can be affected by measurement environments, behavior of subjects, measurement procedures, devices used for measurements and observers [47]. To minimize errors in blood pressure measurement, a standardized measurement with mercury sphygmomanometer was use in our study. The controlled hypertension is not only affected by the blood pressure measurement but also by the cut-off blood pressure value. The cut-off value selected in this study is widely used worldwide. These values are based on the evidence of a meta-analysis from 1994 showing that above these values patients benefit from antihypertensive therapy [48]. The effects of health insurance on blood pressure and control lags behind. After one and a half years of coverage, the outpatient copayment scheme for hypertension might not show significant effects on blood pressure in our study. HRQOL and self-reported health are both self-reported indicators that represent different ways of measuring health. HRQOL assesses how an individual’s well-being (including physical, emotional, and social aspects) may be affected by a disease, disability, or disorder over time [49]. In our study, HRQOL was measured by the EQ-5D. The number of response categories of summary items of the EQ-5D are often few and may be too crude to measure small but important changes in health. Self-reported health referred to both a single question about overall health and a survey questionnaire in which participants assessed different dimensions of their own health [50]. The answer to the self-rated health question is based on what people think and thus is subjective. Self-reported health would be expected to be more sensitive to intervention by insurance providers than HRQOL. Beyond measures of health status, there are many links between outpatient copayment schemes and health outcomes that may limit our ability to demonstrate the effects of health insurance on patient health. These potential events include access to chronic care, prescription of appropriate medications, adherence to medical recommendations, and effectiveness of treatment in improving health [51].

Our study has several limitations. First, antihypertensive drug use was self-reported, and no information about the quality and continuity of treatment was available. Sustained improvements in health require access to quality care, a continuous supply of guideline-based drugs, and adherence to treatment regimens [29, 32, 41, 42]. Second, the insurance of participants in the outpatient copayment scheme for hypertension was not randomized. The insured group had higher overall income than the older, uninsured group (composed primarily of retired patients with a median age of approximately 70). The older, uninsured group also had more severe hypertension, so the findings based on this group are not likely to be generalizable to the entire adult hypertensive population. Third, we only selected one area for research, which did not represent the entire Xinjiang. The research findings cannot be generalized to all of Xinjiang. The Eighth Division would be a good example of the effect of an outpatient copayment scheme on health outcomes of hypertension in Xinjiang. Finally, we only investigated the two-year impact of insurance policies on patients. The survey was conducted 6 and 18 months after the policy changed in 2016. Many studies and systematic reviews found that the longer an insurance programme has been in place prior to the timing of the evaluation, the higher the odds of improved health outcomes [18, 52]. Health effects may take a long time to appear. The patient’s health may not have changed significantly in short time. Therefore, the survey data of sixth month after the policy changed was used as a baseline. And few settings exist where in health insurance programs could be rolled out in a randomized fashion. The outpatient copayment scheme in China required patients to apply voluntarily and determined their eligibility based on the severity of their respective illnesses. We used an alternative approach to eliminate this selection bias by including a control group and analyzing the data using a difference-in-differences propensity score matching method. Future research should investigate the long-term effects of insurance on health in our study population.

## Conclusion

A decreased outpatient copayment scheme was associated with an increase in antihypertensive treatment coverage and an improvement in self-reported health in hypertensive populations in Xinjiang, China. The outpatient copayment scheme was not associated with improvements in blood pressure, EQ5D, controlled hypertension after 18 months. Our study highlighted the potential of outpatient copayment schemes to reduce or eliminate the cost of medications for a community-managed population with hypertension and, in turn, improve their treatment of hypertension. Further consideration should be given to raising the level of reimbursement for outpatient copayment schemes in order to combat the increasing costs of outpatient services.

## Supporting information

S1 File The outpatient copayment scheme descriptions and the changes in insurance. (DOC)

S2 File The results of propensity scores matching. (DOC)

S3 File The survey questions or questionnaire in both the original language and English. (DOC)

S1 Ethics statement (PDF)
