# Supplementary material for: Effect of an outpatient copayment scheme on health outcomes of hypertensive adults in a community-managed population in Xinjiang, China
Source: PLoS One. 2020 Sep 11;15(9):e0238980. doi: 10.1371/journal.pone.0238980 (PMC7485825; doi:10.1371/journal.pone.0238980)
Supplement: S1 File — (DOC) [file pone.0238980.s001.doc]

**Supplementary Materials**

**Contents**

- **The annual data of the prevalence of hypertension and disability-adjusted cumulative life expectancy**
- **The outpatient copayment scheme descriptions**
- **The changes in outpatient copayment scheme and inpatient medical insurance**

**The annual data of the prevalence of hypertension and disability-adjusted cumulative life expectancy**

| Country | Prevalence of hypertension (2014) | Disability-adjusted cumulative life expectancy (2015) | |
| --- | --- | --- | --- |
|  |  | Cardiovascular disease (CVD) | Stroke |
| China | 19.80% | 5,716 | 2,828 |
| the Republic of Korea | 14% | 2,654 | 1,271 |
| Singapore | 16% | 2,866 | 682 |
| the United States | 17% | 4,535 | 966 |

**The outpatient copayment scheme descriptions**

**Information about the outpatient copayment scheme**

The outpatient copayment scheme refers to chronic diseases that meet the requirements. Its application process has four steps: the first is that the patient chooses the designated medical institution and fills in the application materials; the second is that the selected designated medical institution collects the corresponding information of the applicant, and organizes the medical experts of the hospital to check and preliminarily identify; The third is that the Medical Insurance Office organizes the city's urban workers' basic medical insurance expert committee to conduct centralized evaluation, and the expert committees conduct individual verification according to the special confirmation criteria. The fourth is to issue special medical records for eligible patients.

**The identification of hypertension in outpatient copayment scheme.**

**[Identification Criteria]**

More than one year of history of treatment or diagnosis of hypertension, due to long-term high blood pressure and cause damage to the target organs of the heart, brain, kidney, blood vessels, that is, concurrent with one or more of the following 2-7 complications, can be consistent with the identification of chronic diseases of hypertension standard:

(1) More than one-year history of treatment or diagnosis of hypertension;

(2) Hypertensive retinopathy ≥ grade III, fundus photography showing flaming hemorrhage, flocculation, special conditions refer to ophthalmoscopy or fundus fluorescein;

3) Cardiac mild hypertrophy or left ventricular hypertrophy (Electrocardiogram, X-ray shows left ventricular hypertrophy or left heart enlargement, cardiac color Doppler shows left ventricular posterior wall and ventricular septum ≥ 12mm);

4) Initiation of coronary heart disease, a history of hypertension, and the disease has reached the criteria for the identification of chronic diseases of coronary heart disease (except for occult and angina);

(5) Renal damage: 24-hour microalbuminuria or renal dysfunction in proteinuria. Microalbumin test microprotein ≥200mg /24h during hospitalization (requires continuous measurement 3 times, 2 positive cases); serum creatinine ≥121 (urine routine), 24-hour urine protein quantification, early kidney damage, etc.);

(6) Combined cerebrovascular accident (cerebral infarction or cerebral hemorrhage) or cerebral angiography stenosis degree ≥70% (computed tomography, magnetic resonance imaging and other imaging evidence); magnetic resonance imaging showed point-like ischemic lesions;

(7) Hypertension triggers a mezzanine aneurysm.

**[Identification Data]**

(1) Electrocardiogram, stereo electrocardiogram, dynamic blood pressure check report, etc.;

(2) B ultrasound, fundus photography or fundus fluorescence report;

(3) Chest, heart or skull CT or MRI report form;

(4) Relevant clinical test indicators when renal function damage.

**The changes in outpatient copayment scheme and inpatient medical insurance**


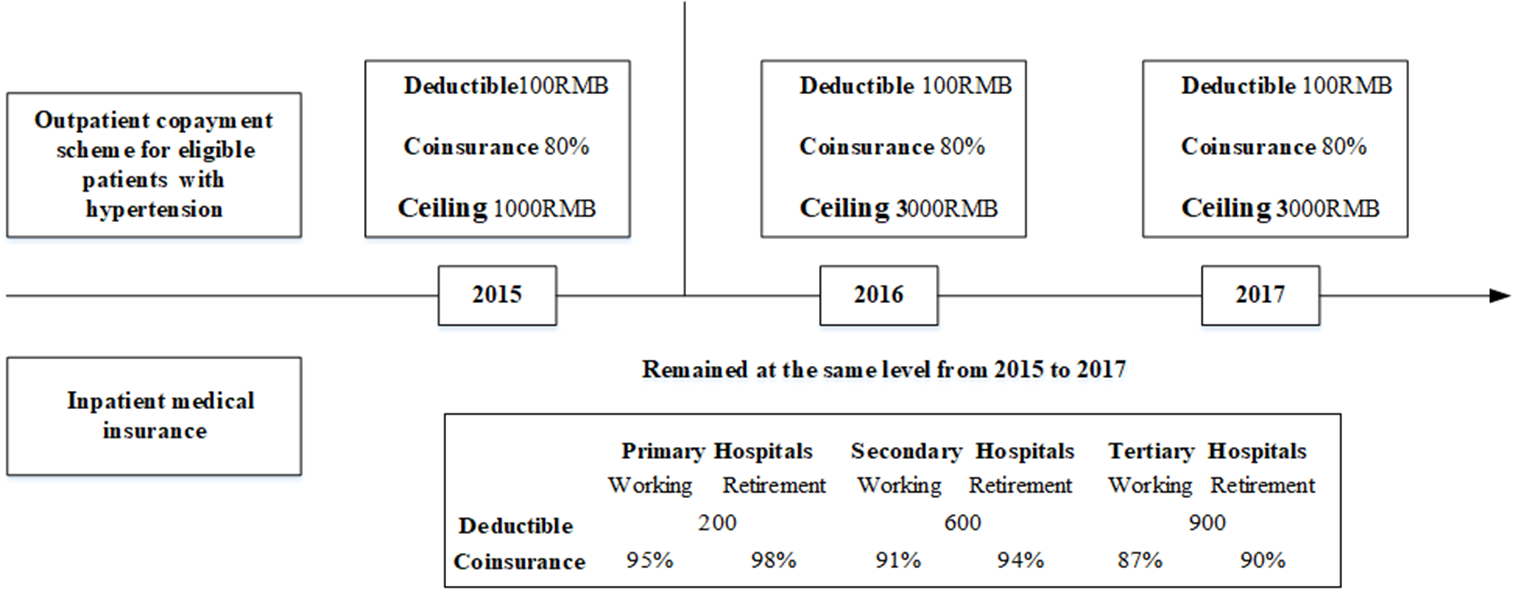


**S1 Fig 1. Changes in outpatient copayment scheme and inpatient medical insurance from 2015 to 2017.**
