# Supplementary material for: Effect of an outpatient copayment scheme on health outcomes of hypertensive adults in a community-managed population in Xinjiang, China
Source: PLoS One. 2020 Sep 11;15(9):e0238980. doi: 10.1371/journal.pone.0238980 (PMC7485825; doi:10.1371/journal.pone.0238980)
Supplement: S2 File — (DOC) [file pone.0238980.s002.doc]

**Supplementary Materials**

**Contents**

- **The results of propensity scores matching**

**The results of propensity scores matching**

**S2 Table 1 The logistic regression for propensity scores**

| Insurance | Coef. | Std. Err. | z | P>z | [95% Conf. Interval] | |
| --- | --- | --- | --- | --- | --- | --- |
| Age | 0.01 | 0.01 | 0.70 | 0.49 | -0.02 | 0.04 |
| Sex | -0.35 | 0.19 | -1.87 | 0.06 | -0.72 | 0.02 |
| Income | 0.13 | 0.14 | 0.99 | 0.32 | -0.13 | 0.40 |
| Marital status | 0.02 | 0.09 | 0.23 | 0.82 | -0.15 | 0.19 |
| Education level | 0.00 | 0.08 | 0.06 | 0.96 | -0.16 | 0.17 |
| Employed | 0.28 | 0.27 | 1.04 | 0.30 | -0.25 | 0.81 |
| Family members | 0.08 | 0.09 | 0.85 | 0.39 | -0.10 | 0.26 |
| Self-reported disease severity | 0.69 | 0.13 | 5.36 | 0.00 | 0.44 | 0.94 |
| Course of hypertension | 0.57 | 0.20 | 2.91 | 0.00 | 0.19 | 0.96 |
| Number of medications | 0.94 | 0.24 | 3.90 | 0.00 | 0.47 | 1.42 |
| _cons | -4.29 | 1.26 | -3.42 | 0.00 | -6.76 | -1.83 |

Number of patients = 749; LR chi2(10) = 76.83;

Prob > chi2 = 0.00; Pseudo R2 = 0.08

**S2 Table 2. The region of common support**

| Estimated propensity score | | |
| --- | --- | --- |
| Percentiles | | Smallest |
| 1% | 0.07 | 0.06 |
| 5% | 0.11 | 0.07 |
| 10% | 0.14 | 0.07 |
| 25% | 0.21 | 0.07 |
| 50% | 0.33 | Largest |
| 75% | 0.42 | 0.72 |
| 90% | 0.50 | 0.73 |
| 95% | 0.57 | 0.74 |
| 99% | 0.67 | 0.76 |

**S2 Table 3 The ATT and ATE with each health outcome**

| Variable | Sample | Uninsured | Insured | Difference | S.E | T-stat |
| --- | --- | --- | --- | --- | --- | --- |
| SBP | Unmatched | 139.40 | 139.54 | 0.14 | 1.28 | 0.11 |
|  | ATT. ^a^ | 140.52 | 139.54 | -0.98 | 1.49 | -0.65 |
|  | ATE ^b^ |  |  | 0.01 |  |  |
| DBP | Unmatched | 80.16 | 79.80 | -0.36 | 0.90 | -0.40 |
|  | ATT | 80.15 | 79.80 | -0.35 | 1.12 | -0.31 |
|  |  |  |  | -0.51 |  |  |
| EQ-5D | Unmatched | 0.89 | 0.84 | -0.05 | 0.01 | -4.30 |
|  | ATT | 0.89 | 0.84 | -0.05 | 0.01 | -3.31 |
|  | ATE |  |  | -0.52 |  |  |
| EQ-VAS | Unmatched | 68.40 | 64.20 | -4.21 | 1.40 | -2.99 |
|  | ATT | 67.43 | 64.20 | -3.23 | 1.72 | -1.88 |
|  | ATE |  |  |  |  |  |
| Contorl | Unmatched | 0.40 | 0.41 | 0.01 | 0.04 | 0.23 |
|  | ATT | 0.39 | 0.41 | 0.02 | 0.04 | 0.56 |
|  | ATE |  |  | 0.00 |  |  |
| Treatment | Unmatched | 0.90 | 0.98 | 0.07 | 0.02 | 3.55 |
|  | ATT | 0.97 | 0.98 | 0.00 | 0.01 | 0.28 |
|  | ATE |  |  | 0.09 |  |  |
| Self-reported health | Unmatched | 0.37 | 0.29 | -0.09 | 0.04 | -2.30 |
|  | ATT | 0.37 | 0.29 | -0.08 | 0.04 | -1.84 |
|  | ATE |  |  | -0.09 |  |  |

Note :S.E. does not take into the propensity score is account that the propensity score is estimated.

^a^: indicates the average treatment effects on the treated

^b^: indicates the average treatment effects

**S2 Table 4. A before/after difference-in-mean test for estimated propensity-score**

| Variable | Mean | | Bias | t-test | | V(T)/V(C) |
| --- | --- | --- | --- | --- | --- | --- |
|  | Insured | Uninsured |  | t | p>t |  |
| propensity-score | 0.39 | 0.38 | 4.9 | 0.58 | 0.56 | 1.17 |

**S2 Table 5. A before/after difference-in-mean test for all the covariates**

| Variable | Mean | | Bias | T-test | | V(T)/  V(C) |
| --- | --- | --- | --- | --- | --- | --- |
|  | Insured | Uninsured |  | t | p>t |  |
| Age | 73.28 | 73.54 | -3.8 | -0.43 | 0.67 | 0.66 |
| Sex | 1.55 | 1.59 | -7.5 | -0.82 | 0.41 | 1.02 |
| Income | 1.66 | 1.66 | 0.6 | 0.06 | 0.95 | 0.92 |
| Marital status | 2.52 | 2.58 | -5 | -0.56 | 0.58 | 0.93 |
| Education level | 2.21 | 2.19 | 1.9 | 0.20 | 0.84 | 0.86 |
| Employed | 2.97 | 2.96 | 2.5 | 0.32 | 0.75 | 0.67 |
| Family members | 2.33 | 2.33 | 0.00 | 0.00 | 1.00 | 0.92 |
| Self-reported disease severity | 0.26 | 0.24 | 4.9 | 0.50 | 0.62 | 1.1 |
| Course of hypertension | 1.74 | 1.78 | -5.8 | -0.84 | 0.40 | 1.21 |
| Number of medications | 1.08 | 1.05 | 8.8 | 1.03 | 0.30 | 1.26 |

**The distribution of the propensity-score for insured and uninsured group before and after Matching.**

**The distribution of the propensity-score for insured and uninsured group before Matching.**


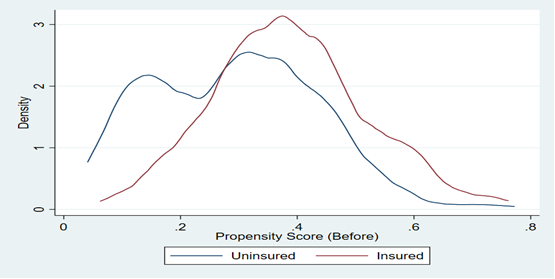


**S2 Fig 1 Distribution of insured and uninsured group before matching**

**The distribution of the propensity-score for insured and uninsured groups after Matching.**

**
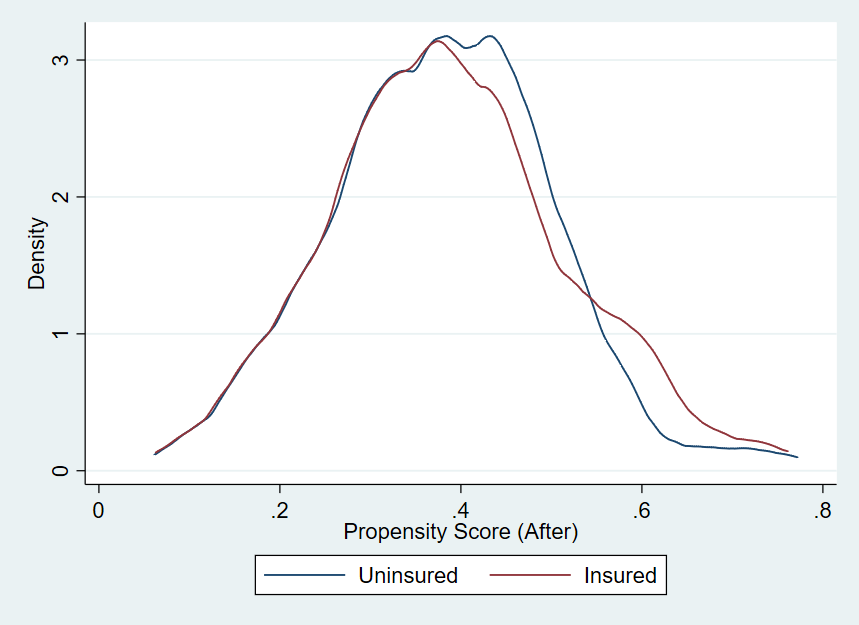
**

**S2 Fig 2 Distribution of insured and uninsured group after matching**
