# Supplementary material for: Effect of an outpatient copayment scheme on health outcomes of hypertensive adults in a community-managed population in Xinjiang, China
Source: PLoS One. 2020 Sep 11;15(9):e0238980. doi: 10.1371/journal.pone.0238980 (PMC7485825; doi:10.1371/journal.pone.0238980)
Supplement: S3 File — (DOC) [file pone.0238980.s003.doc]

**Supplementary Materials**

- **The survey questions or questionnaire in both the original language and English**

**The survey questions or questionnaire in both the original language and English.**

**问卷编码(ID)**  □□----□□□----□□----□□□□----□□ 高血压合并并发症鉴定:是□ 否□

师/市 团 连队卫生室/社区 个人编码 随访次数

**兵团参加基本医疗保险高血压患者就医行为调查表**

调查员致辞：

尊敬的受访者：您好！我们是“石河子市参加基本医疗保险高血压患者就医行为调查课题”的调查员。此次调查旨在了解高血压患者的健康行为及其卫生服务利用的情况，为完善基本医疗保险慢性病补偿政策提供依据，希望得到您的配合。关于被访者信息保密的保证：对于您提供的一切信息，我们会严格遵守《中华人民共和国统计法》予以保密；您的姓名、地址和电话记录仅作为日后随访调查之用，请您无须有任何顾虑。如果您同意我们的调查，请您在此签字 _____________。

姓名(Name)：____________ 联系电话(Tel)：____________

身份证号(Card)：**□□□□□□□□□□□□□□□□□□**

家庭住址(Adr)：____师(Adr1)___团场/城市(Adr2)___连队/社区(Adr3)___门牌号(Adr4)

调查员姓名：__________ 调查员编码(SP)：__________

审核人姓名：__________ 审核人编码：__________

调查日期(Date)：_____/___/___

建档时间(Time):_____/___/___

**表A.家庭及个人一般情况调查表**

| A1 | 您家共有几口人？(过去半年内常住人数，包括非本户人员及新生儿) |  |
| --- | --- | --- |
| A2 | 2015年您家的实际年总收入是多少钱？(包括政府补助) |  |
| A3 | 性别：(1)男 (2)女 |  |
| A4 | 出生年月(年/月) |  |
| A5 | 您目前参加的医疗保险是（可多选）：(1)公费医疗 (2)城镇职工基本医疗保险 (3)城镇居民基本医疗保险 (4)新型农村合作医疗　(5)商业医保 (6)其他_____ |  |
| A6 | 目前婚姻状况：(1)未婚 (2)已婚并共同生活 (3)已婚但分居 (4)离婚 (5)丧偶 (6)其他____ |  |
| A10 | 文化程度：(1)不识字/识字很少(2)小学(3)初中(4)高中或中专(5)大学专科 (6)大学本科 (7)硕士及以上 |  |
| A11 | 从业状况：(1)在业 (2)无业(跳问至表B) (3)离退休 (4)失业(跳问至表B) (5)其他____ |  |

**B.疾病严重程度相关问题**

| B1 | 您高血压的诊断时间？(年，例如2010年) |  |
| --- | --- | --- |
| B2 | 在哪里首次诊断的？(注明_________________)(1)私人诊所 (2)连队卫生室/社区卫生服务站 (3)团医院 (4)社区卫生服务中心 (5)师级(地区市)医院 (6)兵团(省)级医院 (7)中医院 (8)民营医院 (9)其它  ____ |  |
| B3 | 目前您高血压的严重程度为？(1)没有自觉症状 (2)轻度不适，不影响正常工作生活 (3)中度不适，部分工作不能胜任，但不影响日常起居 (4)不能工作，只能生活自理 (5)极度不适，需卧床 |  |
| B4 | 患高血压后，医生是否要求您进行药物治疗? (1)是 (2)否（跳问到B10） |  |
| B5 | 患高血压后，您是否进行药物治疗? (1)是 (2)否(跳问到B10) |  |
| B6 | 如果采用药物治疗，您目前服用的药物，第一种是： |  |
| B7 | 第二种是： |  |
| B8 | 第三种是： |  |
| B9 | 其他： |  |

**表C.个人健康状况及健康意识表**

| B1 | 与2015年相比，您认为自己今年的健康状况有什么变化？(1)没变化 (2)变好了 (3)变坏了 (4)不太好说 |  |
| --- | --- | --- |
| B2 | 今天您在行动方面：(1)可以四处走动，没有任何困难 (2)行动有些不方便 (3)不能下床活动 |  |
| B3 | 今天您在自我照顾方面：(1)无任何问题 (2)有些问题 (3)无法自己洗脸、刷牙、洗澡或穿衣 |  |
| B4 | 今天您从事日常活动方面：(1)从事日常活动没有任何问题 (2)有些问题 (3)无法从事日常活动 |  |
| B5 | 今天您身体疼痛或不舒服方面：(1)无任何疼痛或不舒服(2)有中度疼痛或不舒服(3)有极度疼痛或不舒服 |  |
| B6 | 今天您在焦虑或抑郁方面：(1)没有焦虑或抑郁 (2)有中度焦虑或抑郁 (3)有极度焦虑或抑郁 |  |
| B7 | 请您说出最能代表您今天健康状况好坏的那个分值  “VAS 标尺” |  |
| B8 | 在医务人员的指导和自身配合下，您的血压是否得到了有效地控制？(血压值_____/_____)(1)是 (2)否 |  |

**Questionnaire code ( ID ) □□ ---- □□ ---- □□ ---- □□□□ ---- □□**

**Division / city Group Community Personal code Number of follow-ups**

**Identification of complications associated with hypertension : Yes □ No □**

**Questionnaire on Health outcomes of Hypertensive Patients in Basline**

The investigator addressed:

Dear interviewee: We are the investigator of the project called Investigation on Medical Behavior of Hypertensive Patients with Basic Medical Insurance in Shihezi Xinjiang. The purpose of this survey is to understand the health behavior of hypertensive patients and the utilization of health services and their health outcomes, to provide the basis for improving the basic medical insurance compensation policy for chronic diseases, and hope to get your cooperation. The guarantee of confidentiality of the respondent's information: We will strictly abide by the "Statistics Law of the People's Republic of China" for confidentiality; your name, address and telephone records are only used for future follow-up investigation, please do not need to Any concerns. If you agree to our investigation, please sign here:

Name: ____________________________

ID (Card):**_ □□□□□□□□□□□□□□□□□□**

Family address (Adr): ___ division (Adr1)___ group yard/city (Adr2)___ company/community (Adr3)___ door number (Adr4): ___

Name of investigator: _________________________ Reviewer name: ________________________

Date of investigation:___/_/__ Time for archiving:___/_/__

**Table A.**  **Patient’s’ demographics**

| A1 | How many people are there in your family?(The number of permanent residents in the past six months, including non-residents and newborns) |  |
| --- | --- | --- |
| A2 | What is the actual annual total income of your family in 2015? (including government subsidies) |  |
| A3 | Gender: (1) Male (2) Female |  |
| A4 | Year of birth (Year/Month) |  |
| A5 | The medical insurance you are currently participating in is (optional): (1) Public Medical Care (2) Urban Employee Basic Medical Insurance (3) Urban Resident Basic Medical Insurance (4) New Rural Cooperative Medical Care (5) Commercial Medical Insurance (6) Other__ |  |
| A6 | Current marital status: (1) unmarried (2) married and living together (3) married but separated (4) divorced (5) widowed (6) other___ |  |
| A7 | Education level: (1) illiteracy/little literacy (2) primary school (3) junior high school (4) senior high school or technical secondary school (5) College (6) undergraduate (7) master degree or above |  |
| A8 | Employment status: (1) in-service (2) unemployment (skip to Table B), (3) retirement (4) unemployment (skip to Table B), (5) other___ |  |

**Table B.** **Clinical Characteristics**

| B1 | When did you diagnose hypertension? (Year, for example, 2010) |  |
| --- | --- | --- |
| B2 | Where was the first diagnosis? (noted _________________) (1) private clinic (2) company health room / community health service station (3) group hospital (4) community health service center (5) division level (regional city) hospital (6) Corps (province ) hospital (7) Chinese medicine hospital (8) private hospital (9) other____ |  |
| B3 | What is the current severity of your hypertension? (1) No symptoms (2) Mild discomfort, does not affect normal working life (3) Moderate discomfort, some work is not competent, but does not affect daily life (4) Can't work, can only take care of themselves (5) Extreme discomfort Need to stay in bed |  |
| B4 | Does your doctor require you to take medication after suffering from hypertension? (1) Yes (2) No (jump to B10) |  |
| B5 | After suffering from hypertension, do you take medication? (1) Yes (2) No (jump to B10) |  |
| B6 | If you take medication, the first type of medication you are currently taking is: |  |
| B7 | The second is: |  |
| B8 | The third is: |  |
| B9 | Other: |  |

**Table C. Health outcomes**

| C1 | How do you think your health has changed this year compared with 2015? (1) unchanged (2) better (3) worse (4) not very good to say |  |
| --- | --- | --- |
| C2 | Today in mobility: (1) can move around without any difficulty; (2) some inconveniences in action; (3) can't get out of bed. |  |
| C3 | Today self-care: (1) no problems (2) some problems (3) unable to wash, brush, bathe or dress by yourself. |  |
| C4 | Today in daily activities: (1) there are no problems in daily activities; (2) there are some problems; (3) unable to engage in daily activities. |  |
| C5 | Today in pain or discomfort: (1) no pain or discomfort (2) moderate pain or discomfort (3) extreme pain or discomfort. |  |
| C6 | Today in anxiety or depression: (1) no anxiety or depression (2) moderate anxiety or depression (3) extreme anxiety or depression. |  |
| C7 | Please tell me the score that best represents your health today.  "VAS Scale" |  |
| C8 | Is your blood pressure effectively controlled under the guidance and cooperation of medical staff? (Blood pressure value ___/______) (1) Yes (2) No |  |

**问卷编码(ID)**  □□----□□□----□□----□□□□----□□ 高血压合并并发症鉴定:是□ 否□

师/市 团 连队卫生室/社区 个人编码 随访次数 是否同时患有糖尿病：是□ 否□

**兵团参加基本医疗保险高血压患者就医行为随访表**

调查员致辞：

尊敬的受访者：您好！我们是“石河子市参加基本医疗保险高血压患者就医行为调查课题”的调查员。此次调查旨在了解高血压患者的健康行为及其卫生服务利用的情况，为完善基本医疗保险慢性病补偿政策提供依据，希望得到您的配合。关于被访者信息保密的保证：对于您提供的一切信息，我们会严格遵守《中华人民共和国统计法》予以保密；您的姓名、地址和电话记录仅作为日后随访调查之用，请您无须有任何顾虑。如果您同意我们的调查，请您在此签字_____________。

姓名(Name)：____________联系电话(Tel1)：____________联系电话(Tel2)：____________

身份证号(Card)：**□□□□□□□□□□□□□□□□□□**

家庭住址(Adr)：____师(Adr1)___团场/城市(Adr2)___连队/社区(Adr3)___门牌号(Adr4)

调查员姓名：__________调查员编码(SP)：__________

审核人姓名：__________审核人编码：__________

调查日期(Date)：_____/___/___

建档时间(Time):_____/___/___

**表A.家庭、个人一般情况及健康状况调查表**

| A1 | 您家共有几口人？(过去半年内常住人数，包括非本户人员及新生儿) |  |
| --- | --- | --- |
| A2 | 2016年您家的实际年总收入是多少钱？(包括政府补助) |  |
| A3 | 您目前参加的医疗保险是（可多选）：(1)公费医疗(2)城镇职工基本医疗保险(3)城镇居民基本医疗保险(4)新型农村合作医疗(5)商业医保(6)其他_____ |  |
| A4 | 目前婚姻状况：(1)未婚(2)已婚并共同生活(3)已婚但分居(4)离婚(5)丧偶(6)其他____ |  |
| A5 | 从业状况：(1)在业(2)无业(跳问至A9)(3)离退休(4)失业(跳问至A9)(5)其他____ |  |
| A6 | 您是否申请过高血压病合并并发症慢性病鉴定？(1)是(2)否（跳问至表B）,原因___________ |  |
| A9 | 您是否通过了高血压病合并并发症慢性病鉴定？(1)是(2)否,原因___________ |  |

**表B.疾病严重程度相关问题**

| B1 | 与2016年相比，您认为自己今年的健康状况有什么变化？(1)没变化(2)变好了(3)变坏了(4)不太好说目前您高血压的严重程度为？(1)没有自觉症状 (2)轻度不适，不影响正常工作生活 (3)中度不适，部分工作不能胜任，但不影响日常起居 (4)不能工作，只能生活自理 (5)极度不适，需卧床 |  |
| --- | --- | --- |
| B2 | 目前您高血压的严重程度为？(1)没有自觉症状 (2)轻度不适，不影响正常工作生活 (3)中度不适，部分工作不能胜任，但不影响日常起居 (4)不能工作，只能生活自理 (5)极度不适，需卧床 |  |
| B3 | 患高血压后，医生是否要求您进行药物治疗? (1)是 (2)否（跳问到B9） |  |
| B4 | 患高血压后，您是否进行药物治疗? (1)是 (2)否(跳问到B9) |  |
| B5 | 如果采用药物治疗，您目前服用的药物，第一种是： |  |
| B6 | 第二种是： |  |
| B7 | 第三种是： |  |
| B8 | 其他： |  |
| B9 | 在医务人员的指导和自身配合下，您的血压是否得到了有效地控制？(血压值_____/_____)(1)是 (2)否 |  |

**表C.个人健康状况及健康意识表**

| B1 | 与2016年相比，您认为自己今年的健康状况有什么变化？(1)没变化 (2)变好了 (3)变坏了 (4)不太好说 |  |
| --- | --- | --- |
| B2 | 今天您在行动方面：(1)可以四处走动，没有任何困难 (2)行动有些不方便 (3)不能下床活动 |  |
| B3 | 今天您在自我照顾方面：(1)无任何问题 (2)有些问题 (3)无法自己洗脸、刷牙、洗澡或穿衣 |  |
| B4 | 今天您从事日常活动方面：(1)从事日常活动没有任何问题 (2)有些问题 (3)无法从事日常活动 |  |
| B5 | 今天您身体疼痛或不舒服方面：(1)无任何疼痛或不舒服(2)有中度疼痛或不舒服(3)有极度疼痛或不舒服 |  |
| B6 | 今天您在焦虑或抑郁方面：(1)没有焦虑或抑郁 (2)有中度焦虑或抑郁 (3)有极度焦虑或抑郁 |  |
| B7 | 请您说出最能代表您今天健康状况好坏的那个分值  “VAS 标尺” |  |
| B8 | 在医务人员的指导和自身配合下，您的血压是否得到了有效地控制？(血压值_____/_____)(1)是 (2)否 |  |

**Questionnaire code (ID) □□ ---- □□ ---- □□ ---- □□□□ ---- □□**

**Division / city Group Community Personal code Number of follow-ups**

**Identification of complications associated with hypertension : Yes □ No □**

**Questionnaire on Health outcomes of Hypertensive Patients in Follow-up**

The investigator addressed:

Dear interviewee: We are the investigator of the project called Investigation on Medical Behavior of Hypertensive Patients with Basic Medical Insurance in Shihezi Xinjiang. The purpose of this survey is to understand the health behavior of hypertensive patients and the utilization of health services and their health outcomes, to provide the basis for improving the basic medical insurance compensation policy for chronic diseases, and hope to get your cooperation. The guarantee of confidentiality of the respondent's information: We will strictly abide by the "Statistics Law of the People's Republic of China" for confidentiality; your name, address and telephone records are only used for future follow-up investigation, please do not need to Any concerns. If you agree to our investigation, please sign here:

Name: ____________________________

ID (Card):**_ □□□□□□□□□□□□□□□□□□**

Family address (Adr): ___ division (Adr1)___ group yard/city (Adr2)___ company/community (Adr3)___ door number (Adr4): ___

Name of investigator: _________________________ Reviewer name: ________________________

Date of investigation:___/_/__ Time for archiving:___/_/__

**Table A. Patient’s’ demographics**

| A1 | How many people are there in your family? (The number of permanent residents in the past six months, including non-residents and newborns) |  |
| --- | --- | --- |
| A2 | What is the actual annual total income of your family in 2016? (including government subsidies) |  |
| A3 | The medical insurance you are currently participating in is (optional): (1) Public Medical Care (2) Urban Employee Basic Medical Insurance (3) Urban Resident Basic Medical Insurance (4) New Rural Cooperative Medical Care (5) Commercial Medical Insurance (6) Other__ |  |
| A4 | Current marital status: (1) unmarried (2) married and living together (3) married but separated (4) divorced (5) widowed (6) other___ |  |
| A5 | Employment status: (1) in-service (2) unemployment (jump to A9) (3) retirement (4) unemployment (jump to A9) (5) other___ |  |
| A6 | Have you ever applied for the identification of hypertension complicated with complications and chronic diseases? (1) Yes (2) No (skip to Table B), the reason______ |  |
| A9 | Have you passed the identification of hypertension complicated with complications and chronic diseases? (1) yes (2) no, reason______ |  |

**Table B. Clinical Characteristics**

| B1 | What is the current severity of your hypertension? (1) No symptoms (2) Mild discomfort, does not affect normal working life (3) Moderate discomfort, some work is not competent, but does not affect daily life (4) Can't work, can only take care of themselves (5) Extreme discomfort Need to stay in bed |  |
| --- | --- | --- |
| B2 | Does your doctor require you to take medication after suffering from hypertension? (1) Yes (2) No (jump to B10) |  |
| B3 | After suffering from hypertension, do you take medication? (1) Yes (2) No (jump to B10) |  |
| B4 | If you take medication, the first type of medication you are currently taking is: |  |
| B5 | The second is: |  |
| B6 | The third is: |  |
| B7 | Other: |  |

**Table C. Health outcomes**

| C1 | How do you think your health has changed this year compared with 2015? (1) unchanged (2) better (3) worse (4) not very good to say |  |
| --- | --- | --- |
| C2 | Today in mobility: (1) can move around without any difficulty; (2) some inconveniences in action; (3) can't get out of bed. |  |
| C3 | Today self-care: (1) no problems (2) some problems (3) unable to wash, brush, bathe or dress by yourself. |  |
| C4 | Today in daily activities: (1) there are no problems in daily activities; (2) there are some problems; (3) unable to engage in daily activities. |  |
| C5 | Today in pain or discomfort: (1) no pain or discomfort (2) moderate pain or discomfort (3) extreme pain or discomfort. |  |
| C6 | Today in anxiety or depression: (1) no anxiety or depression (2) moderate anxiety or depression (3) extreme anxiety or depression. |  |
| C7 | Please tell me the score that best represents your health today.  "VAS Scale" |  |
| C8 | Is your blood pressure effectively controlled under the guidance and cooperation of medical staff? (Blood pressure value ___/______) (1) Yes (2) No |  |
