# Supplementary material for: Effect of an outpatient copayment scheme on health outcomes of hypertensive adults in a community-managed population in Xinjiang, China
Source: PLoS One. 2020 Sep 11;15(9):e0238980. doi: 10.1371/journal.pone.0238980 (PMC7485825; doi:10.1371/journal.pone.0238980)
Supplement: S1 Ethics statement — (PDF) [file pone.0238980.s004.pdf]

石河子大学医学院第一附属医院医学伦理委员会
科研项目申报审查意见

项目名称	医疗保障制度设计对慢性病防控的影响研究<br>----以新疆生产建设兵团为例
申请人	井明霞
伦理审核意见	
井明霞　所申请的项目《医疗保障制度设计对慢性病防控的影响研究--以新疆生产建设兵团为例》经医学伦理委员会全面审查，研究内容不违背医学伦理审查要求，同意该申请人申报本项目。<br><br>石河子大学医学院第一附属医院伦理委员会<br>（科教处代章）<br>日期：2013年3月4日	
